# Vascular Endograft Infection with *Listeria monocytogenes* reated with Surgical Debridement but without Graft Removal

**DOI:** 10.1155/2011/482815

**Published:** 2011-06-27

**Authors:** Beate Tanner-Steinmann, Katia Boggian

**Affiliations:** ^1^Department of Infectious Diseases, Cantonal Hospital Lucerne, 6000 Lucerne 16, Switzerland; ^2^Department of Infectious Diseases, Cantonal Hospital St. Gallen, 9000 St. Gallen, Switzerland

## Abstract

The awareness of *Listeria monocytogenes* as a pathogen in meningitis and bacteremia in immunosuppressed patients is high. We report a case of vascular graft infection due to Listeria monocytogenes as an example of a less well-known manifestation of listeriosis and focus on the possible treatment procedures emphasizing a management with surgical debridement but preservation of the endograft, in contrast to the gold standard treatment of vascular graft infections which consists of a removal of the graft.

## 1. Introduction

The awareness of *Listeria monocytogenes* as a pathogen in meningitis in immunosuppressed or elderly patients is high. Also gastroenteritis, infections in pregnancy or sepsis in neonates are well-known clinical manifestations due to Listeria. However, this pathogen can cause many other types of infection. As an example, we report here the so-far 8th case of vascular graft infection due to Listeria, where in general organisms like Staphylococci, *E. coli,* or Streptococci are found. Our aim is to increase the awareness of Listeria as a pathogen causing other clinical syndromes in immunosuppressed patients over and above meningitis and gastroenteritis, and we focus on the treatment options in prosthetic graft infection due to Listeria reviewing the so far reported 7 cases.

## 2. Case Presentation

**Figure 1:**
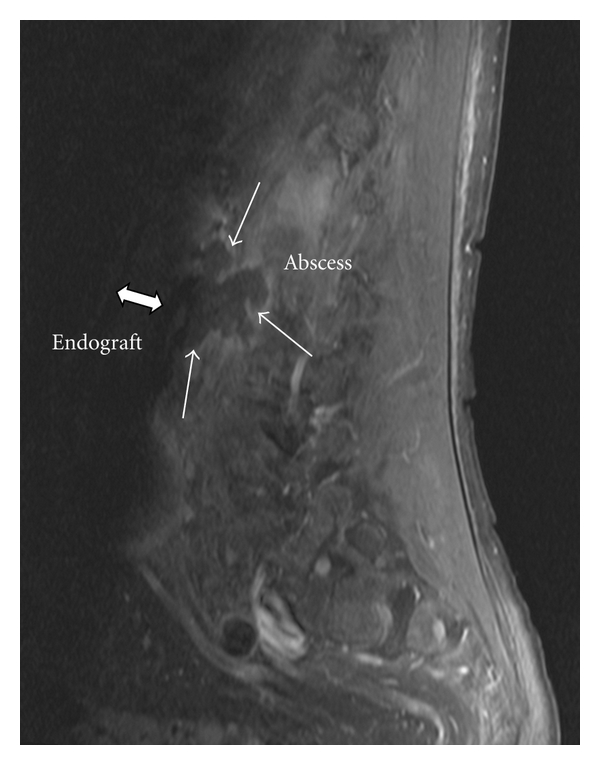
Sagital view of preoperative MRI-scan showing a retroaortal fluid collection with slight enhancement reaching to the dorsal part of the prosthetic endograft.

A 59-year-old man was referred to our hospital in August 2007 because of a progression of his chronic low back pain which began at the beginning of May 2007 accompanied by recurrent episodes of fever. The fever occured a few weeks later at the end of May 2007, and because of a temperature of 39°C the general practitioner treated the patient empirically with a 10-day course of amoxicillin/clavulanate. After that, the fever disappeared for a few weeks, but new episodes of fever and pain attacks occurred in July and August 2007. During this time, the C-reactive protein level was rising to 250 mg/l at admission (standard value <5 mg/l). The patient was under immunosuppression with Ciclosporine A and Mycophenolate-Mofetile after renal transplantation 2004, and he got an endovascular repair of an infrarenal aortic aneurysm with a Talent-graft in 2005. At admission, the patient's low back pain and fluctuating fever were interpreted as a sacroileitis. A MRI-scan showed no skeletal pathology but a slight enhancement of the sclerotic infrarenal aorta including an enhancement of the periaortal tissue. So, a CT guided translumbar needle aspiration of the enhancing structure was taken, and the cultures yielded growth of *Listeria monocytogenes*, whereas the blood cultures remained negative. Therefore, the patient was treated with ampicillin 2 gm q4h intravenously without gentamicin because of his impaired renal function (see Figure 1). 

Under this antibiotic treatment, the CRP-level sank initially, but remained then around 120 mg/l so that gentamicin 1 mg/kg i.v. three times a day was added. At this time, the surgeon refused a surgical approach since the renal transplant was vasculated through the graft and a removal of the graft would have been a high risk for the transplanted organ. After 6 weeks of combined antibiotic therapy a new MRI showed new collections around the vascular graft down to the psoas muscle. A drainage delivered pure pus, the cultures were sterile but an in-house eubacterial PCR (16S ribosomal DNA sequence analysis) was positive for Listeria. So after 2 months of conservative treatment a surgical approach was faced due to lacking improvement. Intraoperatively extensive inflammatory adhesions were found; therefore, the aneurysm with the endograft could not be removed. Only a debridement around the aneurysm and in the psoas muscle could be performed. The Mycophenolate-Mofetil dosing was reduced at that time. Two weeks thereafter and after 8 weeks of combined intravenous antibiotic therapy the patient could be discharged with an oral regimen consisting of 320 mg trimethoprim/1600 mg sulfamethoxazole twice a day. A MRI-scan 6 months later in June 2008 showed no more abscesses but a slight enhancement in the periaortic tissue as well as in the psoas muscle. In January 2009, the CRP achieved a normal level (<5 mg/l), and, in July 2009, there was no more enhancement in the MRI-scan. Nevertheless, we decided to continue the antibiotic treatment for another 2 years (until July 2011) since the patient has a stable function of his renal transplant and since a relapse of the graft infection would be disastrous for the whole situation of the patient. In March 2011, the patient is well without any symptoms.

## 3. Discussion

A prosthetic graft infection is a very rare (0.6% to 3%) but serious complication after aneurysm repair with a high mortality (25% to 88%) [8–10]. Although there are no guidelines, the gold standard in treatment consists of a complete excision of the graft, reconstruction of an extra-anatomic bypass, and an antibiotic treatment for several weeks [8, 11]. Generally, organisms like Staphylococci or gramnegative enteric organisms as *E. coli* are responsible for prosthetic graft infections [9]. Graft infections due to Listeria are extremely rare. Including our case, only 8 cases have been reported [1–7]. In general, *Listeria monocytogenes* is an uncommon cause of severe illness. Healthy hosts may develop self-limited febrile gastroenteritis, whereas patients with risk factors are prone for invasive disease. Risk factors are the extremes of age (neonates and elderly persons >60 years), pregnancy, and especially impaired cellular immunity as in organ transplant recipients or patients with hematologic malignancies [12]. According to that, five of the eight vascular graft infections due to Listeria occurred in patients showing at least one of the reported risk factors like age (case no. 1, 6, 7) or immunosuppression (case no. 3, 8) (see Table 1). Our case is the only case of Listerial graft infection in a solid organ transplanted patient. 

In the 8 analysed cases, with one exception, the mean interval between graft implantation and onset of infection was 2 years (range 12 weeks to 10 years), indicating a hematogeneous spread of infection. Listeria occur ubiquitous are mainly ingested with contaminated food, and invasive disease evolves by hematogeneous spread from the gut [13]. 1–5% of healthy individuals are asymptomatic intestinal carriers of Listeria [12]. This was presumably also the way our patient achieved the graft infection, but it was not possible to determine the source where he ingested Listeria. He did not report having eaten raw milk or soft cheese nor did he purchase food from delicatessen counters regularly. As a chemist, he did not have any risk by profession like, for example, farmers who are in direct contact to cattle which are possible carriers of Listeria as well. Only 5% of all cases of Listeriosis occur in outbreaks. In Switzerland, the last outbreak was in 2005 [14] but in an area far from where the patient lived. Apart from his immunosuppression due to the renal transplantation, our patient did not have any further risk factors for Listeriosis as for example described by Fernàndez-Sebé et al. [15] for solid-organ transplant recipients: no diabetes mellitus, no history of CMV-disease, or high-dose steroid therapy in the preceding 6 months. As reported by Stamm et al. [16], Listeriosis in renal transplant recipients in particular occured in 75% during the first year after transplantation. In our case, the manifestation of the infection was 4 years after transplantation, but the moment of acquisition remains unclear.

The diagnosis of graft infections can be extremely challenging. Symptoms are often nonspecific like recurrent fever and chills or back pain as it was in our case. With a sensitivity of 94% and a specificity of 85% CT-scan is considered the diagnostic test of choice [8, 9], but since it cannot distinguish sterile fluid from perigraft abscedation, PET/CT seems to be a promising method. Blood cultures are only positive in 21%, cultures of drain production or of the prosthesis itself in 50% [8]. Considering the 8 reported cases with Listerial graft infections blood cultures were taken in only 5 of the 8 patients and were positive in three of the five. An aspirate of the perigraft fluid was taken in 6 of the cases, and all of the samples showed growth of listeria. This fact points out that culturing perigraft fluid seems to have a considerably higher sensitivity compared to the 50% reported by Saleem et al. [8].

With regard to the treatment strategy, persistence or recurrence of the infection was observed in both with (case no. 2) and without (cases no. 4–8) initial removal of the prosthesis (see Table 1), where at the gold standard of treatment consists of an early removal of the graft. Antibiotic therapy alone was initially tried in 6 of the 8 cases, but was successful in only two patients (cases no. 3, 4). In one case a percutaneous drainage was finally successful (no. 7), and in our case (no. 8), a surgical debridement without graft removal was successful in the end (see Table 1). So, we conclude and agree with Saleem et al. [7, 8] that in patients with a high perioperative risk, a preservation of the endograft together with a prolonged and high-dose antimicrobial therapy combined with radiological drainage or surgical debridement but without graft removal is a possible treatment option. 

## Figures and Tables

**Table 1:** Summary of the 8 so-far reported graft infections due to *Listeria monocytogenes*.

No	RF	BC	Foc	Early surgery	Follow up	Antibiotics	Ref
1	Age (75 y)	ND	+	Removal of Gore-Tex prosthesis, thrombectomy of dacron cross-over graft	*After 3 weeks:* death from pneumonia	Amox/clavulanic acid i.v. + Gm i.m.	[1]

2	HD	ND	+	Partial removal of graft, PTFE graft interposition	*After 3 months:* recurrence with sepsis → shunt removal, antibiotic therapy not described	CTX 3 × 1 g i.v. 2d; then Amp 4 × 1 g i.v. 7d; then Amp 4 × 500 mg p.o. 4d	[2]

3	Steroids + Azathioprine for several months	+	ND	ND	None	Amp iv 6 × 2 g 4 weeks; then STX po 4 weeks	[3]

4	None	+	ND	ND	*7 weeks after stop antibiotics*: recurrence, conservative treatment	Amp 4 × 2 g i.v. 19 d + Gm 1 × 80 mg i.v. 4d *Recurrence*: Amp 6 × 2 g i.v. 6 weeks + Gm 240 mg i.v. 4 weeks; then Amox 3 × 1 g po 8 weeks	[4]

5	HD, DM	+	+	ND	*2 weeks after stop Vanco: *relapse → graft and abscess removal	Vanco 750 mg/wk for 3 weeks *Relapse:* Amp i.v. 3 weeks	[5]

6	Age (77 y)	−	+	ND	*After 2 weeks of persistence: *resection of aneurysm	Amp iv 6 × 2 g + Net 1 × 300 mg 2 weeks *After resection:* Amp 6 × 2 g i.v. for 6 weeks; then Doxy 150 mg for 4 weeks	[6]

7	Age (67 y), DM	ND	+	ND	*Later:* percutaneous drainage	Amox-clavulanic acid i.v. + STX 800/160 3/d, then STX 6 months	[7]

8	Trspl (Age 59 y)	−	+	ND	*After 6 weeks:* percutaneous drainage and surgical debridement	Amp 6 × 2 g + Gm 1 mg/kg i.v. tid for 6 weeks; *After debridement*: Amp 6 × 2 g + Gm 1 mg/kg i.v. tid for 2 weeks; then STX for 4 years	Tanner

Amox: amoxicillin; Amp, ampicillin; BC: blood cultures; CTX: cefotaxime; DM: diabetes mellitus; Foc: cultures of focus; Gm: gentamicin; HD: hemodialysis, Net: netilmicin; ND: not done; PTFE: polytetrafluoroethylene; RF: risk factors; SXT trimethoprim-sulfamethoxazole; tid: 3 times a day; Tobra: trobramycin; Trspl: transplanation; Vanco: vancomycin.
